# Accumulation of medium-chain, saturated fatty acyl moieties in seed oils of transgenic *Camelina sativa*

**DOI:** 10.1371/journal.pone.0172296

**Published:** 2017-02-17

**Authors:** Zhaohui Hu, Qian Wu, Jyoti Dalal, Naresh Vasani, Harry O. Lopez, Heike W. Sederoff, Rongda Qu

**Affiliations:** 1 Department of Crop and Soil Sciences, North Carolina State University, Raleigh, North Carolina, United States of America; 2 Department of Plant and Microbial Biology, North Carolina State University, Raleigh, North Carolina, United States of America; INRA, FRANCE

## Abstract

With its high seed oil content, the mustard family plant *Camelina sativa* has gained attention as a potential biofuel source. As a bioenergy crop, camelina has many advantages. It grows on marginal land with low demand for water and fertilizer, has a relatively short life cycle, and is stress tolerant. As most other crop seed oils, camelina seed triacylglycerols (TAGs) consist of mostly long, unsaturated fatty acyl moieties, which is not desirable for biofuel processing. In our efforts to produce shorter, saturated chain fatty acyl moieties in camelina seed oil for conversion to jet fuel, a 12:0-acyl-carrier thioesterase gene, *UcFATB1*, from California bay (*Umbellularia californica* Nutt.) was expressed in camelina seeds. Up to 40% of short chain laurate (C12:0) and myristate (C14:0) were present in TAGs of the seed oil of the transgenics. The total oil content and germination rate of the transgenic seeds were not affected. Analysis of positions of these two fatty acyl moieties in TAGs indicated that they were present at the *sn-1* and *sn-3* positions, but not *sn-2*, on the TAGs. Suppression of the camelina *KASII* genes by RNAi constructs led to higher accumulation of palmitate (C16:0), from 7.5% up to 28.5%, and further reduction of longer, unsaturated fatty acids in seed TAGs. Co-transformation of camelina with both constructs resulted in enhanced accumulation of all three medium-chain, saturated fatty acids in camelina seed oils. Our results show that a California bay gene can be successfully used to modify the oil composition in camelina seed and present a new biological alternative for jet fuel production.

## Introduction

*Camelina sativa* (L.) Crtz., is a member of the Brassicaceae family. It is also known as false flax or gold of pleasure, an ancient crop native to Europe and Central Asia [1]. Recently, camelina has gained attention as a potential biofuel crop with many industrial applications in North America and other temperate climate zones of the world [2, 3, 4]. Camelina is tolerant to cold and drought, requires little fertilizer or pest control, and grows well on marginal land where other oil crops such as corn and soybean usually do not grow efficiently [5]. The relatively short life cycle of camelina (100–120 days from planting to harvest) provides another advantage for farmers, because it can grow as a spring or fall rotation crop. It is an ideal crop for improvement through genetic engineering because it can be easily transformed by *Agrobacterium* using floral vacuum infiltration [6].

Camelina seed normally contains 37–42% oils, mostly in the form of triacylglycerols (TAGs). More than 80% of the fatty acyl moieties (FAs) in the TAGs are long-chain (C18 –C22) and unsaturated, which is undesirable as a feedstock for conversion to jet fuel. The main energy-dense component in jet fuel or biodiesel is a mixture of medium-chain hydrocarbons. To make jet fuel or biodiesel from TAGs, after removal of glycerol, the unsaturated FAs need to be hydrogenated and then converted to medium-chain C10-C14 and short-chain C6-C9 hydrocarbons [7]. These processes are costly and energy-consuming. Crop biotechnology has provided both insights and tools to change the FA composition in plant seed oils [8–10]. The major goal of this project is to alter camelina seed oils by increasing the medium-chain saturated FAs and reducing the abundance of long-chain, unsaturated FAs in TAGs in order to reduce the processing energy, cost and carbon loss during refining to jet fuel.

In general, temperate plants like camelina contain little or no medium-chain FAs, such as laurate (C12:0) in their seed TAGs. However, the seed oil of the temperate California bay tree (*Umbellularia californica* Nutt., belonging to the family of Lauraceae) contains up to 70% laurate with much of the remaining fatty acids being capricate (C10:0). FA synthesis in plants is carried out through a series of enzymes in the plastids. Fatty acid synthases sequentially condense two carbon units onto the growing fatty acyl chain, and the final products are generally C16:0, C18:0 and C18:1 FAs. Thioesterases catalyze the removal of newly formed acyl groups from acyl-acyl carrier protein (ACP) and release them from the plastid FA synthetic pathway. The released FAs could be incorporated into storage oils. The 12:0-acyl-carrier protein thioesterase gene isolated from California bay (*UcFATB1*) encodes a thioesterase, which specifically releases medium-chain FAs, like C12:0 and C14:0, to be incorporated into TAGs. This gene has been successfully introduced in *Arabidopsis thaliana* and *Brassica napus* to redirect FA biosynthesis from mainly C18 unsaturated FAs to largely laurate [9, 10]. In our research, we introduced *UcFATB1* into camelina and expressed it specifically in maturing seeds driven by a seed-specific *napin* gene promoter [9].

β-ketoacyl-acyl-carrier protein synthases (KAS) are the condensing enzymes that extend growing fatty acyl chains. KAS enzymes are present in plastids and catalyze the stepwise condensation of an acyl group bound either to an acyl carrier protein (ACP) or a CoA with malonyl-ACP. Three classes of distinct plant plastid KAS enzymes (KASI, KASII and KASIII) with different acyl substrate specificities have been described, and their corresponding genes cloned [11, 12]. Each type of KAS is responsible for condensation reactions with certain chain-length fatty acids [13]. KASIII initiates fatty acid biosynthesis by condensing acetyl-CoA and malonyl ACP while KASI elongates the acyl chain 2 C at a time to C12:0 –C16:0. KASII functions primarily in stearate (C18:0) synthesis and is most active with 14:0 or 16:0 as a substrate. Down-regulation of *KASII* increases palmitate (C16:0) and reduces the abundance of longer-chain (≥ C18) and/or desaturated FAs in Arabidopsis seed oils [12].

In this paper, we report expression of the *UcFATB1*gene alone or together with camelina *CsKASII* RNAi constructs in transgenic camelina, and demonstrate the accumulation of medium-chain, saturated fatty acyl moieties in camelina seed oil for biodiesel and jet fuel use. Two decades ago, the Calgene group showed successes in using *UcFATB*1 to drastically change the long to medium acyl moieties of seed TAGs in transformed *Brassica napus* [9, 10]. After we have initiated the experiments in this report, another lab [14] reported the use of different *Cuphea FATB* genes, and also *UcFATB*1, to be equally successful in modifying camelina seed oils. Our findings using California bay *UcFATB1* and camelina *CsKASII* RNAi constructs confirm earlier studies in this area [12, 14] and further illustrate the versatility of the modification systems (growth condition, transformation protocol, species varieties, gene promoters, insertion sites, transformed lines, etc.). This illustrated versatility allows us to predict that similar modifications for the biofuel as well as the polymer industries are achievable with genes from other species, such as elm (C8 and C10) [15], palm kernel (C12), and meadowfoam (C22) [16], and recipient species other than *Brassica* and *Camelina*.

## Materials and methods

### Plant materials

Camelina (*C*. *sativa* cv. Calena) plants were grown in a greenhouse at 22℃ under natural light conditions supplemented with LED light with a 12 h photoperiod (12 h of light and 12 h of dark). Plants were watered once a day and fertilized with Osmocote^®^ (The Scotts Company, Marysville, OH) periodically.

### Vector construction and camelina transformation

The *UcFATB1*(NCBI GI:170555) coding sequence from California bay under control of a seed-specific *napin* gene promoter [9] was commercially synthesized (GenScript, Piscataway, NJ) and cloned into the binary vector mCherry-pCAMBIA2300, which contains an mCherry reporter gene as a screenable marker [17].

*C*. *sativa* is a hexaploid and has three KASII family members. For RNAi design, two constructs were created: *RNAi1* is an RNAi construct containing a DNA fragment (169 bp) from the 5’UTR of a *CsKASII* gene (NCBI GI: 408384463, Table A in S1 File) under control of the *β-phaseolin* gene promoter [12]. The sequence has 99% sequence identity to another *CsKASII* gene (NCBI GI: 408384465). The third *CsKASII* gene (NCBI GI: 408384467) has a short reported 5’ UTR sequence and has 94% identity with 49 bp of the 169 bp fragment. *RNAi2* has an RNAi construct of a DNA fragment of 194 bp coding sequence from a *CsKASII* gene (GI: 408384467, Table A in S1 File), with 92% and 96% sequence identity with two other *KASII* genes (GI: 408384463 and GI: 408384465), respectively, driven by the *napin* promoter [9]. Both constructs used the *AtFAD2* first intron [12, 18] as a linker between the two reversely oriented DNA fragments and the *Nos* terminator in the binary vector pCAMBIA2300 (Figure A in S1 File).

The screenable marker *mCherry* was driven by the CaMV 35S promoter in all constructs. The binary vector was introduced into *Agrobacterium tumefaciens* strain GV3101 [6] by electroporation in 0.2 cm cuvettes (Bio-Rad, Hercules, CA) at a field strength of 2.5 kv/cm, resistance of 600 Ω and a capacitance of 25 microF.

Vacuum infiltration transformation was performed with camelina inflorescences as described by Lu et al. [6]. Co-transformation was performed using two *Agrobacterium* strains in 1:1 ratio. Transgenic seeds were identified by *mCherry* reporter gene expression using fluorescence microscopy [17].

### Molecular analysis of transgenic plants

To verify the presence of the transgenes in the mCherry positive plants, PCR was performed on genomic DNA of selected plants. DNA was isolated from leaves using the Shorty buffer (0.2 M Tris-HCl, pH 9.0, 0.4 M LiCl, 25 mM EDTA and 1% SDS, doi:10.1101/pdb.rec11660, Cold Spring Harbor Protocol 2009). To investigate the expression of the transgenes, RNA was isolated from T_3_ individual immature seeds (15–20 days after pollination) of transgenic and wild type (WT) plants using the Quick-RNA™ MiniPrep kit (Zymo Research, Irvine, CA). cDNA was synthesized using the ImProm-II™ Reverse Transcription System (Promega, Madison, WI). *UcFATB1* or *CsKASII* gene expression was measured by qRT-PCR using the SYBR Green method (iTaq™ Universal SYBR^®^ Green supermix, Bio-Rad) with the MX3005P qPCR system (Agilent Technologies, Santa Clara, CA). The primer sequences are listed in Table B in S1 File. For *CsKASII* transcript detection, the primers were designed in a way that they can amplify the coding sequences of all the three *CsKASII* genes, and yet won’t amplify *CsKASI* and *CsKASIII*, the two related genes.

For Southern analysis, total genomic DNAs were isolated from leaves of transgenic and wild-type (Wt) plants. Genomic DNAs were digested with *Bam*HI (New England Biolabs, Ipswich, MA), which has a single cleavage site within T-DNA section of the construct, but not within the *UcFATB1* coding sequence (Figure B in S1 File). The digestion products of each line were electrophoresed through a 1% (w/v) agarose gel, and blotted onto a positively charged nylon membrane (Amersham Hybond™-N^+^, GE Healthcare Life Sciences, Pittsburgh, PA) as described in DIG Application Manual for Filter Hybridization (Roche Diagnostics GmbH, Mannheim, Germany). The DIG labeled probe, specific to *UcFATB1* gene was synthesized using PCR DIG Probe Synthesis Kit. All labeling, hybridization and detection were carried out based on DIG Application Manual for Filter Hybridization (Roche). All labeling, hybridization and detection reagents were purchased from Sigma-Aldrich (St. Louis, MO). Hybridization was carried out at 42°C overnight. Low and high stringent washes were performed as described in DIG Application Manual for Filter Hybridization (Roche). The chemiluminiscent substrate CSPD was used in detection, according to manufacturer’s instructions (Roche). The signal was detected on an X-ray film.

### Total fatty acid composition analysis of transgenic seeds

Seeds were placed directly into glass tubes and fatty acids were converted to Fatty Acid Methyl Esters (FAMEs) by heating at 90℃ in 1 ml 2.5% H_2_SO_4_ (v/v) in methanol for 90 min. FAMEs were extracted with 200 microliter hexane and 1.5 ml 0.5% NaCl (w/v). After vortexing and centrifuging at 3,000 g for 3 minutes, the hexane phase was transferred to auto-injector vials. FAMEs were analyzed by gas chromatography (GC, Agilent 6890 gas chromatograph, Agilent Technologies, Wilmington, DE) with flame ionization detection. Resolution of FAMEs was achieved with an HP-INNOWax column (30 m length, 0.25 mm inner diameter). The oven temperature was programmed to increase from 190℃ to 200℃ at a rate of 12℃/min, held for 3 min at 200℃, then increased to 250℃ at 30℃/min and held for 6.5 min at 250°C.

### Characterization of TAGs and phosphatidylcholines (PCs)

Total lipids were extracted from mature seeds on ice as described [19, 20]. In brief, 50 seeds were mixed with 3 ml chloroform: methanol (1:2, v/v) and ground with mortar and pestle. Ground seeds and solvent were transferred to a 10 ml glass tube and the mixture made up to 5.8 ml by the addition of 1 ml chloroform and 1.8 ml of 0.9% NaCl, so that the final relative volume of chloroform: methanol: NaCl was 1:1:0.9 (v/v/v). The chloroform phase was transferred to a clean glass tube and evaporated to dryness under a stream of N_2_. Lipids were re-suspended in a small volume of chloroform and stored at -20℃.

TAGs were separated from total lipid extracts by TLC (silica gel G60 plates, EM Separations Technology, Gibbstown, NJ) with hexane: diethyl ether: acetic acid (140:60:2, v/v/v) as the developing solvent [21]. Phosphatidylcholines were resolved by TLC of the total lipids with a solvent system consisting of CHCl_3_: MeOH: H_2_O: 30% ammonium hydroxide (65:35:3:2.5, v/v/v/v) [22]. Lipid bands were visualized under UV light after staining with 0.01% primulin in 80% acetone and were scraped from the TLC plate into glass tubes. FAMEs were prepared using transmethylation and analyzed by GC as described above in “Total fatty acid composition analysis of transgenic seeds” section.

### Sn-2 analysis of TAGs

Neutral lipids were separated by TLC as described above. TAG fractions were eluted from the silica gel with a small volume of chloroform/ methanol (1:2, v/v), adding 0.9% NaCl and centrifuging the mixture in a glass tube. The chloroform phase (TAGs) was transferred to a clean glass tube and dried under N_2_. The fatty acid composition of the sn-2 position of TAGs was determined from sn-2 monoacylglycerols (MAG) generated by lipase digestion of the TAGs. The positional analysis of TAG was conducted using *Rhizopus arrhizus* lipase (Sigma, St. Louis, MO) as described by Bafor et al. [23]. Half to one mg of TAG was resuspended in 1 mL of diethyl ether and 0.8 mL of buffer containing 0.1 M-Tris/HCl, pH 7.7, 5 mM CaCl_2_ and 1,200 units of *Rhizopus arrhizus* lipase. The mixture was incubated at room temperature for 2–3 h with vigorous shaking. After removal of the diethyl ether with a stream of N_2_, the lipids were extracted as described by Bligh & Dyer [19]. Lipase hydrolysis products (from the CHCl_3_ layer) were separated by TLC on silica gel G60 plates with hexane/diethyl ether/acetic acid (70:140:3, v/v/v) [21, 24]. For fatty acid analysis, lipid fractions of MAG and TAG species were scraped from the TLC plate into glass tubes and FAMEs were prepared and analyzed by GC.

### Seed oil content

Seed oil content measurements were measured by following the method of Li et al. [25] with minor modifications. Thirty T_4_ homozygous seeds or WT seeds were used for measuring oil contents and fatty acid profiles. Glyceryl triheptadecanoate (Sigma, St. Louis, MO) was used as a TAG internal standard. One milliliter of 2.5% (v/v) sulfuric acid in methanol was added to each sample in glass tube and kept at 90°C for 90 min. The FAs were quantified as methyl esters and analyzed by gas chromatography as described above.

### Seed germination

Fifty seeds per line were sown directly in soil (Fafard growing mix, Sun Gro Horticulture, Agawam, MA) in the greenhouse as described above. Radicle emergence was recorded four days later as seed germination.

## Results and discussion

### Effects of the *UcFATB1* transgene on camelina seed oil composition

A construct containing the *UcFATB1* gene, encoding a 12:0-acyl-carrier protein thioesterase from California bay, under control of the promoter of the *Brassica napus* seed storage protein *napin* gene [9, 10], was introduced into *Camelina sativa* (cv. Calena) via *Agrobacterium*-mediated transformation using floral dip plus vacuum infiltration approach [6]. With the fluorescent *mCherry* gene as a screenable marker at seed stage [17], ten independent transgenic events were obtained. T_2_ seeds of seven *UcFATB1* transgenic camelina lines and WT were analyzed for FA composition in their seed oil. Six mature seeds expressing *mCherry* from each line were pooled for oil analysis by GC. The results showed that *UcFATB1* expression greatly enhanced the accumulation of laurate (C12:0) in seed oil, ranging from 16.6% in Line 80 to 34.4% (molar percentage) in Line 12, compared to no detection in WT (Table 1). In addition, a few percent of myristate (C14:0) was also generated, indicating that UcFATB1 has hydrolytic activities on both acyl-ACPs with a strong preference for C12:0-ACP over C14:0-ACP. The molar percentage of C12:0 plus C14:0 fatty acyl moieties in seed oil from the transgenic plants ranged from 18.5% to 38.3%. Together with C16:0, the medium-chain, saturated fatty acyl moieties were as high as 43%, as shown in Line 12 (Table 1). Accumulation of C12:0 and C14:0 in transgenic seed oil reduced the levels of C16:0 and C18:0 due to direct competition for the substrates. In addition, two other fatty acyl moieties reduced by even larger extent: α-linolenic acid (C18:3) content reduced nearly half, from 37.8% to 19.1–24.3%, and C20:1 decreased in a similar margin, from 14.1% to 4.8–9.9%. Interestingly, the contents of C18:1 and C18:2 were little affected (Table 1), indicating a tight regulation of the metabolic flux in TAG biosynthesis.

**Table 1:** Fatty acid compositions (mol%) of seed lipids in UcFATB1-transgenic camelina and in the wild type. T2 mCherry positive seeds from 7 independent transgenic lines and wild type were analyzed by gas chromatography. Values are the means±SD from five biological replicates. nd: not detectable.

Lines	12:0	14:0	16:0	18:0	18:1	18:2	18:3	20:0	20:1	22:1
Wild type	nd	nd	7.0±0.2	4.5±0.1	14.9±0.4	17.0±0.5	37.8±1.5	1.6±0.1	14.1±0.3	3.2±0.1
4	32.2±2.1	3.9±0.2	6.0±0.2	2.1±0.3	9.0±0.3	16.7±1.0	20.5±0.9	1.9±0.2	5.4±0.1	2.3±0.0
12	34.4±1.5	3.8±0.1	4.8±0.1	2.6±0.2	10.0±0.2	16.5±0.6	19.2±0.6	2.0±0.2	4.8±0.2	2.0±0.1
63	30.1±2.8	3.0±0.1	5.8±0.2	2.4±0.1	12.9±0.8	17.3±0.3	19.1±2.0	2.3±0.1	5.3±0.2	2.0±0.2
75	22.7±0.9	3.2±0.2	5.4±0.3	2.9±0.4	12.7±0.5	18.2±0.3	22.0±2.3	1.6±0.1	8.7±0.3	2.6±0.0
80	16.6±0.9	2.3±0.1	6.3±0.4	2.8±0.1	12.6±0.3	20.6±1.1	24.0±1.6	1.8±0.3	9.9±0.2	3.1±0.3
81	24.0±1.8	2.8±0.3	7.3±0.3	1.9±0.2	10.9±0.5	17.2±0.3	24.3±2.6	1.7±0.0	6.9±0.2	2.9±0.1
82	26.4±1.9	3.8±0.2	7.8±0.2	2.1±0.3	10.4±0.3	16.8±1.2	22.3±1.3	2.2±0.2	5.6±0.1	2.7±0.1

**Fig 1:**
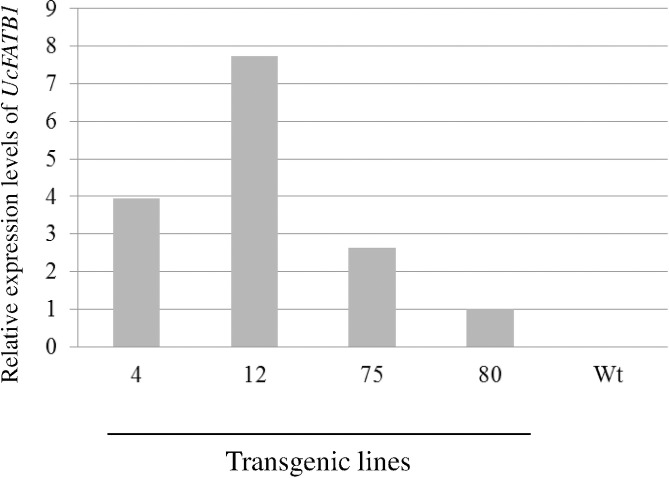
Transgene *UcFATB1* expression was detected in T_3_ immature seeds. There is no *UcFATB1* transgene expression detected in Wt. The *UcFATB1* expression level of Line 80 was used as a calibrator (expression level was arbitrarily set at 1), other lines’ expression levels were expressed as the fold of the expression level of line 80.

Four transgenic lines were randomly chosen to analyze *UcFATB1* expression in transgenic maturing seeds by qRT-PCR. The relative transgene expression levels varied by 8 fold, with Line 12 being the highest (Fig 1). The analysis revealed a general trend: the higher the transgene expression, the higher contents of C12:0 and C14:0 in seed TAGs (Fig 1, Table 1).

To address the question whether higher transgene expression was caused by transgene copy number or by position effect, Southern analysis was performed. In the Southern blot, Line 12 appeared to have simpler hybridization pattern and less transgene copy number than Lines 4 and 75 (Figure B in S1 File), indicating that the high transgene expression in Line 12 is most likely due to position effect rather than higher transgene copy number, although we cannot exclude the possibility that partial transgene silencing took place in Lines 4 and 75 due to multiple transgene copies.

### Incorporation of C12:0 in PCs is low in *UcFATB1* transgenic camelina seeds

The fatty acid chains are synthesized up to C18 with no or only partial desaturation within the plastid. Once exported out of the plastid, newly synthesized acyl-CoAs could incorporate into phosphatidylcholines (PCs) as structural components of the endoplasmic reticulum (ER) membrane lipids or substrates for further desaturation, or elongation, and finally formation of diacylglycerols (DAGs) and TAGs. Identification of fatty acyl moieties composition in PCs is a powerful tool to understand the metabolic pattern of FAs [26].

**Fig 2:**
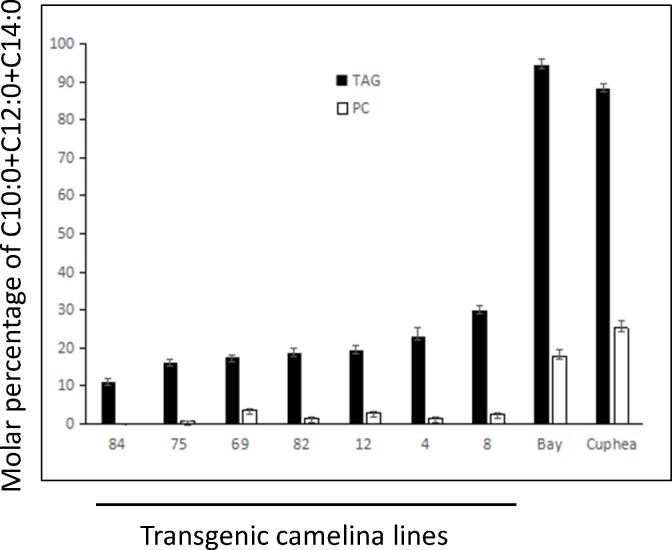
Distribution of C10:0, C12:0 and C14:0 FAs in TAGs and PCs of T_3_ mCherry positive seeds from seven *UcFATB1* transgenic camelina lines, and California bay and *Cuphea wrightii*. Values are the means ± SD from three biological replicates, 50 seeds per rep.

We extracted total lipids from the *UcFATB1* transgenic seeds, separated TAGs and PCs by thin layer chromatography (TLC), and analyzed the FA composition from each class by GC. Whereas C12:0+C14:0 increased from 11.1 to 30.0% in transgenic seed TAGs, C12:0+C14:0 in seed PC remained at low levels (below 3.5%) in transgenic seed oil (Fig 2), suggesting that PCs were not competing for medium-chain fatty acid incorporation to form TAGs. In other words, C12:0- and C14:0-acyl-CoAs are less likely to incorporate into PC permanently but more likely to incorporate into TAG molecules. California bay and *Cuphea wrightii* have high contents of medium-chain FAs in both TAGs and PCs in their seeds (Fig 2). The percentage of C10:0 and C12:0 together in their PCs reached 17.9% and 25.2%, respectively, demonstrating that their seeds incorporated more medium-chain FAs (C10:0+C12:0) into membrane lipids. These results show a difference in the lipid metabolism between camelina and plants naturally enriched with medium-chain fatty acids, like California bay and Cuphea.

### C12:0 and C14:0 were not incorporated at the *sn-2* position of TAG

Seed storage TAGs are synthesized in the ER with acyl-CoA and glycerol-3-P as substrates via the Kennedy pathway [27], in which acylation takes place sequentially from *sn*-1 to *sn*-3 by various enzymes. Among the three positions, incorporation of the medium-chain-length acyl group into *sn*-2 could be strictly controlled by specific enzymes. For example, lysophosphatidic acid acyltransferase (LPAAT) from coconut (*Cocos nucifera*) endosperm can incorporate medium-chain-length acyl group specifically at this position but the rapeseed LPAAT cannot do so [28]. In rapeseed, expression of the California bay *UcFATB1* gene led to C12:0 accumulation but the C12:0 was exclusively incorporated into the *sn*-1 and *sn*-3 positions of the glycerol backbone [10, 28], limiting the total laurate level in transgenic seed oil.

To investigate whether it is the case in the oil of *UcFATB1* transgenic camelina seeds, we analyzed *Rhizopus arrhizus* lipase digested TAGs from T_3_ homozygous seeds of transgenic line 12. *Rhizopus arrhizus* lipase has high selectivity for deacylating the acyl residues located at the *sn*-1 and *sn*-3 positions of TAGs. The *sn*-2 monoacylglycerols (*sn*-2 MAG) produced by the lipase reaction could be separated by TLC, and the acyl residues esterified at the *sn*-2 position of MAG could be determined by GC. As shown in Table 1, whereas ~30% of C12:0 was incorporated into the transgenic camelina seed oils, it was completely absent from the *sn*-2 position of the TAGs, indicating that C12:0 was exclusively incorporated at *sn*-1 and/or *sn*-3 positions, and not at *sn*-2 position. When compared with California bay seed TAGs, we found 72.5% of C10:0 FAs and only 3.0% of C12:0 FAs at the *sn*-2 position, although there was more C12:0 (57.2%) than C10:0 (37.2%) accumulated in its TAGs (Table 2). This suggests that California bay likely has an LPAAT using C10:0 as a preferred substrate for *sn*-2 incorporation.

**Table 2:** Fatty acid composition of TAGs and *sn*-2 MAG from *UcFATB1* transgenic camelina T_3_ homozygous line 12 and California bay. Values are the means ± SD of three biological replicates with 50 seeds per rep. (nd: not detectable).

	FAs composition (molar percentage)										
	10:0	12:0	14:0	16:0	18:0	18:1	18:2	18:3	20:0	20:1	22:1
TAGs,transgenic camelina	nd	25.7±1.5	4.4**±**0.6	5.4±0.7	2.5±0.6	9.7±0.9	17.1±1.1	26.8±1.4	nd	8.5±0.9	nd
Sn-2 MAG,transgenic camelina	nd	nd	nd	10.9±1.1	8.1±0.8	60.3±2.9	5.3±0.5	15.4±1.0	nd	nd	nd
TAGs, California bay	37.2±2.0	57.2±2.5	2.3±0.3	nd	nd	2.7±0.2	0.6±0.0	nd	nd	nd	nd
Sn-2 MAG, California bay	72.5±3.0	3.0±0.4	nd	2.9±0.3	1.3±0.1	20.3±1.2	nd	nd	nd	nd	nd

Apparently, camelina does not have an LPAAT that can incorporate medium-chain acyl groups at the *sn*-2 position. This positional limitation is a negative factor in genetic engineering for high 12:0 accumulation in TAGs in transgenic plants. It has been demonstrated that introduction of a LPAAT from coconut helps solve the problem and further increases medium-chain FAs in camelina seed oils [10, 28].

### Oil content and germination of *UcFATB1* transgenic seeds

**Fig 3:**
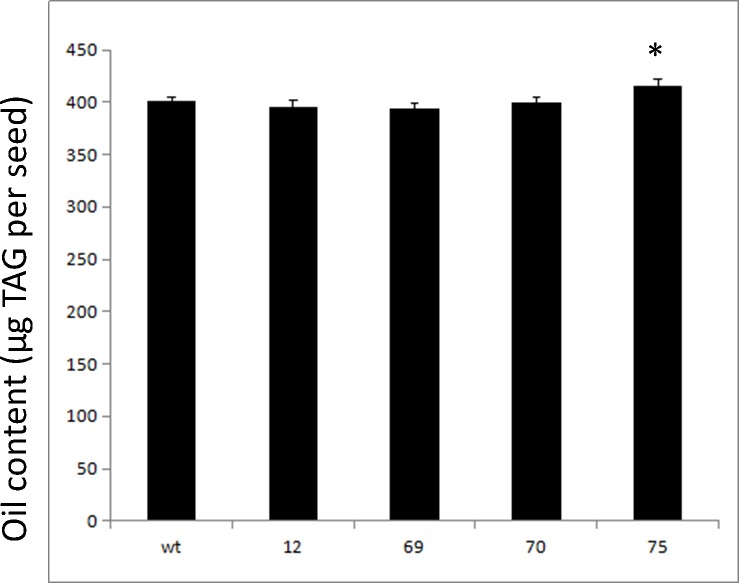
Seed oil content analysis of four T_4_ *UcFATB1* transgenic lines and WT as measured with gas chromatography (30 mCherry positive or WT seeds per sample, 5 replicates). * indicates significant difference compared to other samples (P<0.05).

With the significant FA composition change in *UcFATB1* transgenic seed oil, we further examined the total oil content and seed germination rates from four transgenic lines and wild type plants. The total oil content in seeds of three transgenic lines (Lines 12, 69, and 70) were comparable to WT while transgenic Line 75 had even significantly higher oil content than WT in their seeds (P< 0.05, Fig 3).

Fifty mCherry positive seeds of T_4_ generation from each of the four transgenic lines and wild type plants were subjected to germination tests. The germination rates were above 94% for seeds from all transgenic lines and the wild type. Our data demonstrated that increased content of medium-chain, saturated FAs in seed oils affects neither the total seed oil contents nor seed germination of transgenic camelina.

### Effects of *CsKASII RNAi* on FA composition

Sequences of three annotated *Camelina sativa KASII* gene family members were found in the NCBI database. Two RNAi constructs were designed in an attempt to suppress *CsKASII* gene expression in maturing seeds to increase C16:0 accumulation and to reduce long-chain, unsaturated FAs. The first construct, *RNAi1*, used the French bean *phaseolin* gene promoter and a 169 bp 5’UTR segment of one *CsKASII* gene (NCBI GI: 408384463, Table A in S1 File). This 169 bp segment shares 99% nucleotide identity to the 5’ UTR sequence of another *CsKASII* gene (NCBI GI: 408384465). Due to the short reported 5’UTR on the third *CsKASII* gene (NCBI GI: 408384467) (most likely it is an incomplete-length cDNA), only 49 bp of the 5’ UTR sequence from this gene share 94% identity with the 169 bp fragment used for RNAi construct. The second construct, *RNAi2*, contained a rapeseed *napin* promoter [9] and a 194 bp coding sequence of GI: 408384467, which shares high identity (92% and 96%, respectively) to the corresponding nucleotide sequences of the other two annotated *CsKASII* genes. Suppression of *CsKASII* gene expression in developing seeds of three RNAi1 and one RNAi2 transgenic lines was studied by qRT-PCR (Figure C in S1 File), and substantial increase of C16:0 content in the seed oil was observed in the *CsKASII* RNAi plants. C16:0 increased by 43% to 120% (10.7–16.5 mol% of total FAs) in RNAi1 transgenic lines and by 195% to 280% (22.1% and 28.5 mol% of total FAs) in RNAi2 lines when compared to the WT (7.5 mol%, Table 3). The level of C16:0 increased in *KASII* RNAi transgenic seed oils was similar to what reported in *Arabidopsis* and camelina [12, 14, 29]. The increase of C16:0 in the seed TAGs was compensated by reduction of longer-chain FAs. While in all the cases, C20:1 was reduced by about 40%, and C18:0 was slightly reduced, other FA composition changes varied among transgenic plants. In RNAi2-1 and RNAi2-5, C18:1 was reduced by ~50%, C18:2 roughly stayed the same, and C18:3 was also reduced. In the cases of RNAi1-2 and RNAi1-3, C18:1 stayed the same, C18:3 was further reduced, while C18:2 was unexpectedly doubled when compared to WT. Interestingly, in RNAi1-1, the *CsKASII* transcripts were near the level of WT (Figure C in S1 File), but the C16:0 content was doubled whereas *CsKASII* level in RNAi1-2 was 5 fold less than RNAi1-1, yet the C16:0 content was only increased by 47% (Table 3). A plausible explanation could be somehow the KASII enzyme activities were not correlated with the transcript levels. An enzyme activity assay is needed to prove the hypothesis. Alternatively, *CsKASII* RNAi may interfere the regulation of the TAG biosynthesis, as demonstrated above, and cause variations of the supplies of the FA moieties and their incorporation into TAGs. Overall, the results suggest that suppression of *CsKASII* is an efficient way to increase C16:0 in seed oil. It appears that suppression by RNAi2 construct was more effective than RNAi1.

**Table 3:** Fatty acid compositions of T_3_ mCherry positive seeds from *CsKASII RNAi1* and *CsKASII RNAi2* transgenic lines. Each value is the mean ± SD of five biological replicates, six seeds per rep.

Transgenic lines	Fatty acid composition (molar percentage)							
	16:0	18:0	18:1	18:2	18:3	20:0	20:1	22:1
RNAi1-1	16.5±0.8	2.3±0.3	12.1±0.7	17.5±0.6	36.5±2.1	2.3±0.1	9.7±0.7	3.1±0.3
RNAi1-2	11.0±0.5	3.5±0.2	14.0±0.4	30.1±1.2	27.6±1.5	2.5±0.1	8.5±0.8	2.8±0.5
RNAi1-3	10.7±0.7	3.6±0.4	14.4±0.7	29.3±1.1	28.3±1.3	2.8±0.3	8.3±0.7	2.6±0.3
RNAi2-1	22.1±1.3	2.9±0.1	7.1±0.6	19.3±1.0	33.7±1.8	2.5±0.2	9.5±1.0	2.9±0.5
RNAi2-5	28.5±1.4	3.0±0.2	6.9±1.0	15.4±0.9	32.7±1.9	2.3±0.4	8.2±0.6	3.0±0.2
Wild type	7.5±0.9	4.3±0.5	14.7±0.8	16.9±1.2	37.7±1.7	1.6±0.1	14.1±1.1	3.2±0.1

### Effects of co-transformation with *UcFATB1* and *KASII RNAi2* gene constructs

**Fig 4:**
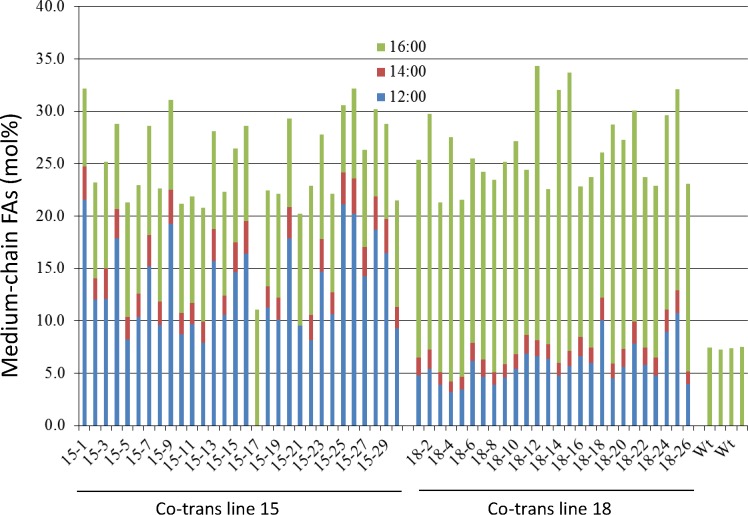
Medium-chain, saturated FA compositions in seed oil of two camelina lines co-transformed with *UcFATB1* and *CsKASII* RNAi-2. Thirty T_2_ seeds of Line 15, and 26 seeds of Line 18, and four wild type seeds were analyzed by gas chromatography.

To maximize medium-chain, saturated FAs in transgenic camelina seed oil, co-transformation was performed by mixing two *Agrobacterium* strains containing either *UcFATB1* or *CsKASII RNAi2* gene constructs. The T_2_ mCherry positive seeds were analyzed for FA compositions in seed oil of the two representative co-transformed lines. Segregation was observed in the 30 seeds analyzed from co-transformation line 15 (Fig 4). Seed No. 15–17 had increased C16:0 but no C12:0 and C14:0 FAs detected, suggesting that this seed had only the *KASII* RNAi construct. Seeds No. 15–1 and 15–25 had accumulated C12:0 and C14:0, but the C16:0 level was similar to that in WT, indicating that these seeds had the *UcFATB1* transgene only. All other examined seeds had both C12:0 and C14:0 detected and C16:0 substantially higher than WT, suggesting the presence of both transgenes. In co-transformed line 18 (Fig 4), all 26 seeds analyzed contained doubled amount of C16:0 compared to that in WT and significant amounts of C12:0 and C14:0, indicating the presence of both transgenes. It appears that in this line, the two transgenes were linked and inherited as one genetic locus, a phenomenon often observed in co-transformation [30]. However, the total content of medium-chain, saturated FAs (C12:0, C14:0 and C16:0) in these co-transformed seeds were roughly between 25–30% of the total FA content in the seed oil, but slightly lower than the highest ones transformed with *UcFATB1* alone (Table 1). Similar phenomenon was reported by Kim et al. (2015) that suppression of *KASII* in camelina increased C16:0 content however decreased C12:0 content in *CsKASII* RNAi/*UcFatB1* lines [14]. This result is most likely due to the limited pool of the substrate supply consumed by the two genetically modified enzymes in the same metabolic pathway: removal of C12:0 from the pathway would reduce the supply for the C16:0 synthesis.

### Concluding remarks

By introducing *UcFATB1* or an RNAi construct of the *CsKASII* genes, we were able to significantly increase the contents of medium-chain, saturated FAs (C12:0, C14:0, and C16:0) in transgenic camelina seed oil. For instance, up to 43 mol% of these three fatty acyl moieties (including 34 mol% of C12:0) in seed oils was observed in *UcFATB1* transgenic camelina (Table 1). When both transgenes are present, over 34 mol% of these three FAs was obtained (with 26 mol% being C16:0, Fig 4). Moreover, we demonstrate that the altered compositions of triacylglycerols do not affect the amount of the total seed oils and seed germination ability.

## Supporting information

S1 File Table A. Sequences used in making *CsKASII* RNAi constructs. Table B. Primers used for gene detection. Figure A. *CsKASII* RNAi constructs. RNAi-1, *β-phas*, *β-phaseolin* gene promoter; *CsKASII 169*, 169 bp 5’UTR sequence of a *CsKASII*. B: RNAi-2, *Napin*, *Napin* promoter; *CsKASII 194*, 194 bp of coding sequence of a *CsKASII*. *AtFAD2 intron*, linker sequence used between two reversely oriented DNA fragments; *Nos*, *nopaline synthase gene* terminator. See details in the text. Figure B. Southern analysis. A. T-DNA section of *UcFATB1* overexpression construct. RB: Right Border; Napin: *Napin* promoter; UcFATB1: California bay 12:0-acyl-carrier protein thioesterase gene (NCBI GI:170555); 35S: cauliflower mosaic virus 35S promoter; mCherry: *mCherry* fluorescence gene; LB: Left Border. BamHI was used for genomic DNA digestion and the probe used in Southern was a fragment of *UcFATB1* coding sequence. B. Southern blot analysis of three independent *UcFATB1* transgenic lines (No. 4, 12 and 75) and a non-transgenic camelina plant (Wt). Figure C. Relative expression of *CsKASII* gene in *CsKASII* RNAi transformed and wild type lines. RNA was extracted from T_3_ individual immature seeds 15–20 days after pollination. Gene expression was measured by qRT-PCR SYBR Green method and normalized to *PP2A* expression.
(PPTX)
