# Supplementary material for: Accumulation of medium-chain, saturated fatty acyl moieties in seed oils of transgenic Camelina sativa
Source: PLoS One. 2017 Feb 17;12(2):e0172296. doi: 10.1371/journal.pone.0172296 (PMC5315392; doi:10.1371/journal.pone.0172296)
Supplement: S1 File — Table A. Sequences used in making CsKASII RNAi constructs. Table B. Primers used for gene detection. Figure A. CsKASII RNAi constructs. RNAi-1, β-phas, β-phaseolin gene promoter; CsKASII 169, 169 bp 5’UTR sequence of a CsKASII. B: RNAi-2, Napin, Napin promoter; CsKASII 194, 194 bp of coding sequence of a CsKASII. AtFAD2 intron, linker sequence used between two reversely oriented DNA fragments; Nos, nopaline synthase gene terminator. See details in the text. Figure B. Southern analysis. A. T-DNA section of UcFATB1 overexpression construct. RB: Right Border; Napin: Napin promoter; UcFATB1: California bay 12:0-acyl-carrier protein thioesterase gene (NCBI GI:170555); 35S: cauliflower mosaic virus 35S promoter; mCherry: mCherry fluorescence gene; LB: Left Border. BamHI was used for genomic DNA digestion and the probe used in Southern was a fragment of UcFATB1 coding sequence. B. Southern blot analysis of three independent UcFATB1 transgenic lines (No. 4, 12 and 75) and a non-transgenic camelina plant (Wt). Figure C. Relative expression of CsKASII gene in CsKASII RNAi transformed and wild type lines. RNA was extracted from T3 individual immature seeds 15–20 days after pollination. Gene expression was measured by qRT-PCR SYBR Green method and normalized to PP2A expression. (PPTX) [file pone.0172296.s001.pptx]

## Slide 1
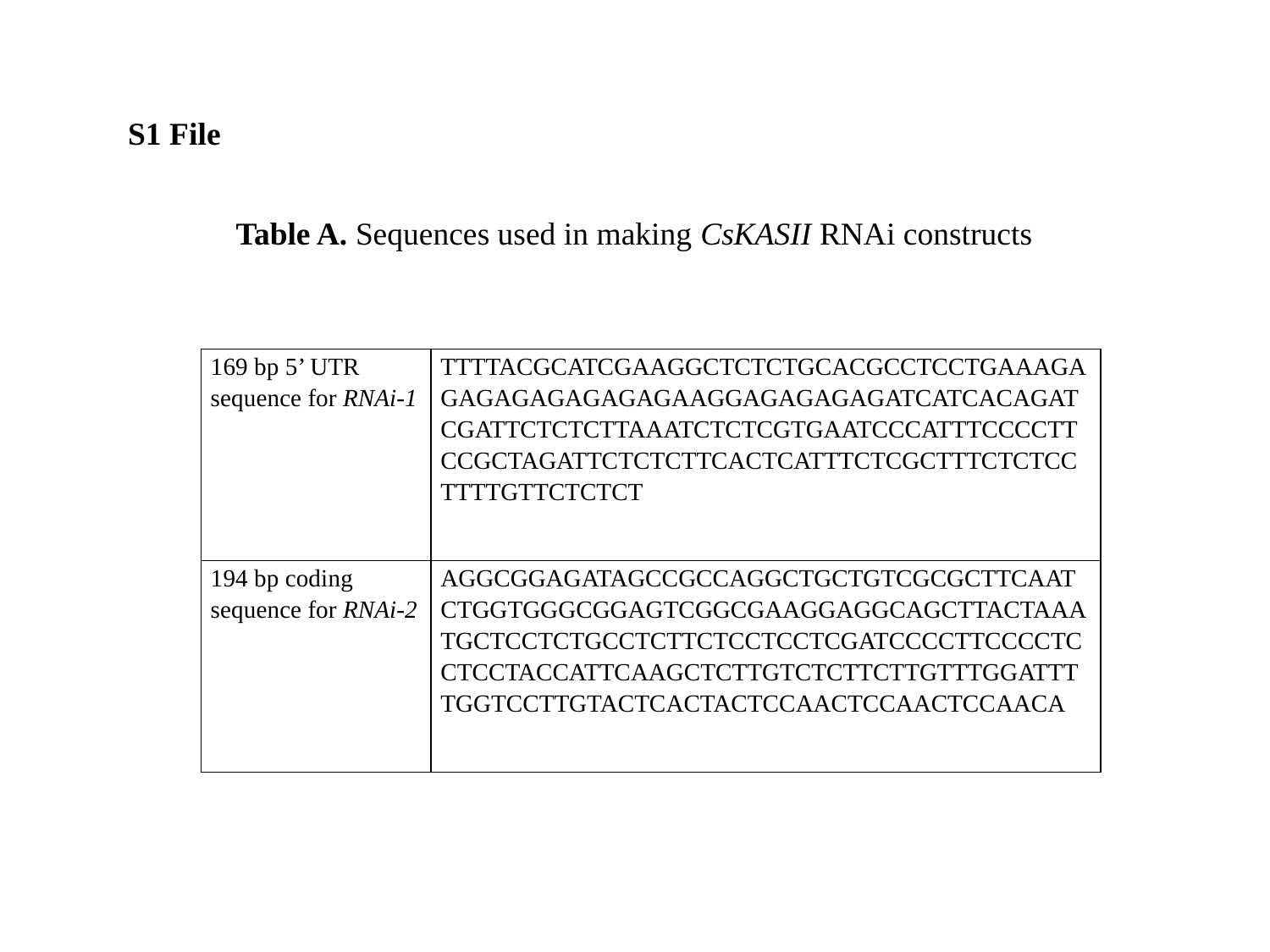

S1 File
Table A. Sequences used in making CsKASII RNAi constructs
| 169 bp 5’ UTR sequence for RNAi-1 | TTTTACGCATCGAAGGCTCTCTGCACGCCTCCTGAAAGAGAGAGAGAGAGAGAAGGAGAGAGAGATCATCACAGATCGATTCTCTCTTAAATCTCTCGTGAATCCCATTTCCCCTTCCGCTAGATTCTCTCTTCACTCATTTCTCGCTTTCTCTCCTTTTGTTCTCTCT |
| --- | --- |
| 194 bp coding sequence for RNAi-2 | AGGCGGAGATAGCCGCCAGGCTGCTGTCGCGCTTCAATCTGGTGGGCGGAGTCGGCGAAGGAGGCAGCTTACTAAATGCTCCTCTGCCTCTTCTCCTCCTCGATCCCCTTCCCCTCCTCCTACCATTCAAGCTCTTGTCTCTTCTTGTTTGGATTTTGGTCCTTGTACTCACTACTCCAACTCCAACTCCAACA |

## Slide 2
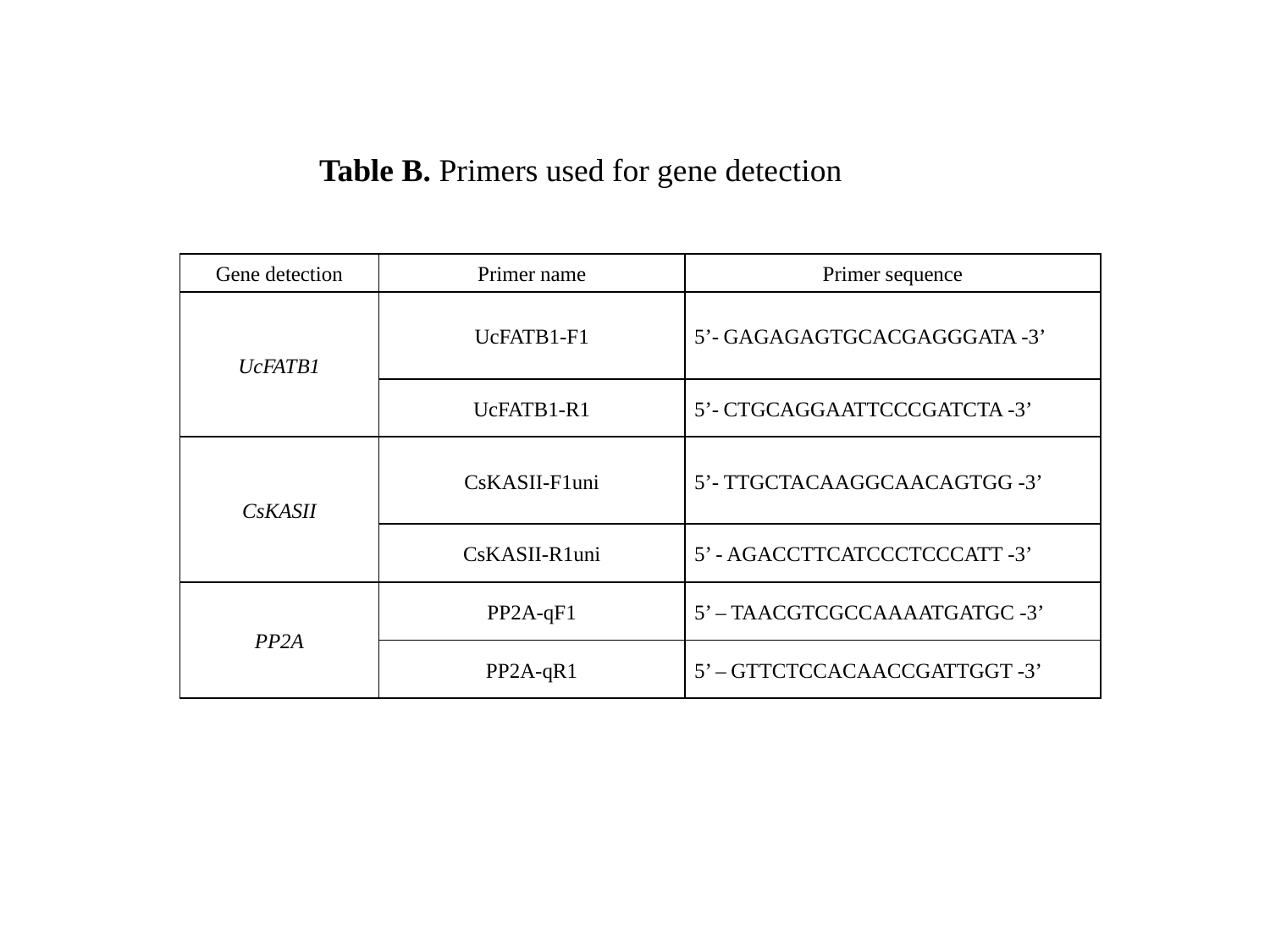

Table B. Primers used for gene detection
| Gene detection | Primer name | Primer sequence |
| --- | --- | --- |
| UcFATB1 | UcFATB1-F1 | 5’- GAGAGAGTGCACGAGGGATA -3’ |
| | UcFATB1-R1 | 5’- CTGCAGGAATTCCCGATCTA -3’ |
| CsKASII | CsKASII-F1uni | 5’- TTGCTACAAGGCAACAGTGG -3’ |
| | CsKASII-R1uni | 5’ - AGACCTTCATCCCTCCCATT -3’ |
| PP2A | PP2A-qF1 | 5’ – TAACGTCGCCAAAATGATGC -3’ |
| | PP2A-qR1 | 5’ – GTTCTCCACAACCGATTGGT -3’ |

## Slide 3
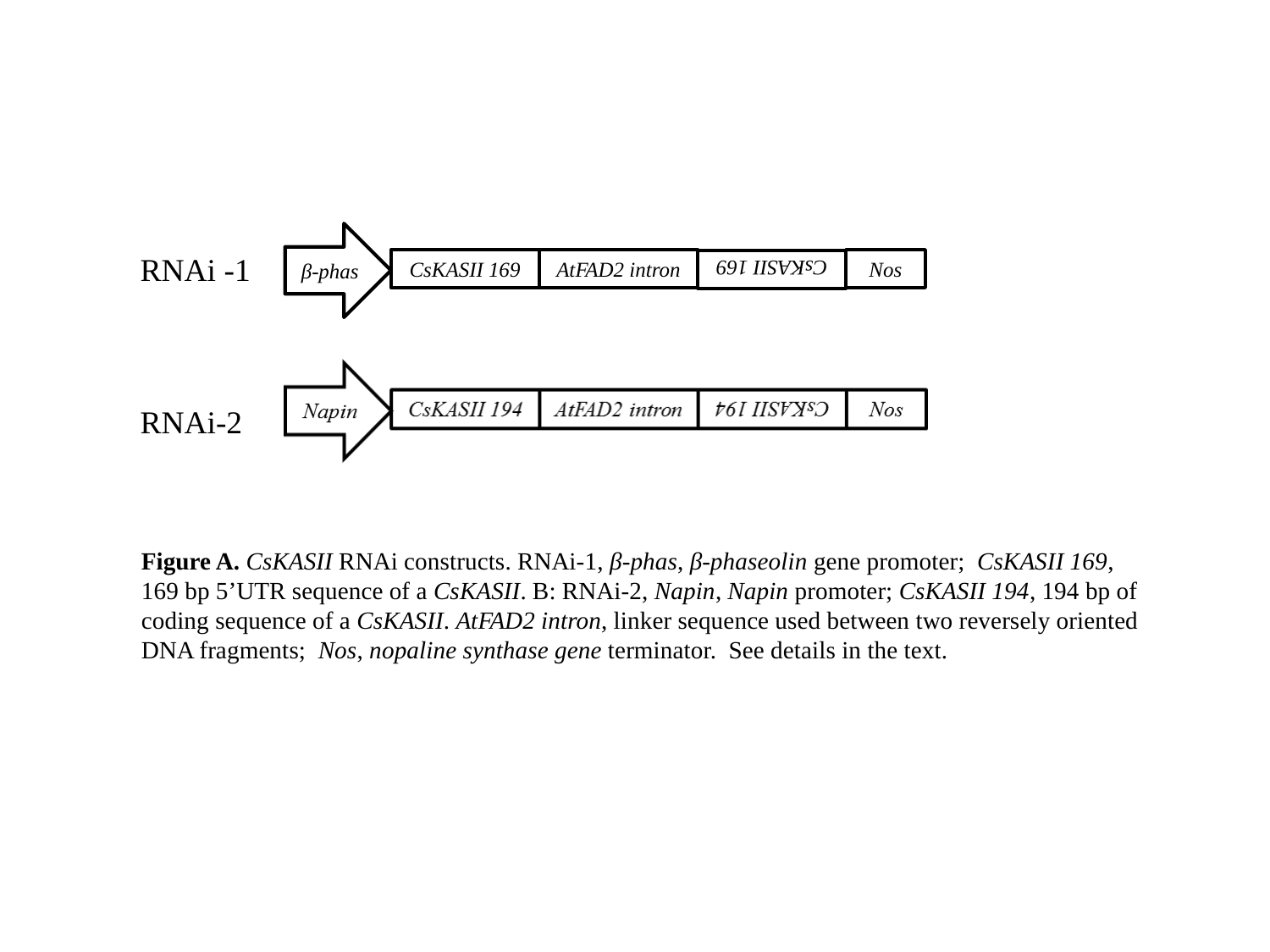

CsKASII 169
AtFAD2 intron
Nos
CsKASII 169
β-phas
RNAi -1
RNAi-2
Figure A. CsKASII RNAi constructs. RNAi-1, β-phas, β-phaseolin gene promoter; CsKASII 169,
169 bp 5’UTR sequence of a CsKASII. B: RNAi-2, Napin, Napin promoter; CsKASII 194, 194 bp of
coding sequence of a CsKASII. AtFAD2 intron, linker sequence used between two reversely oriented
DNA fragments; Nos, nopaline synthase gene terminator. See details in the text.

## Slide 4
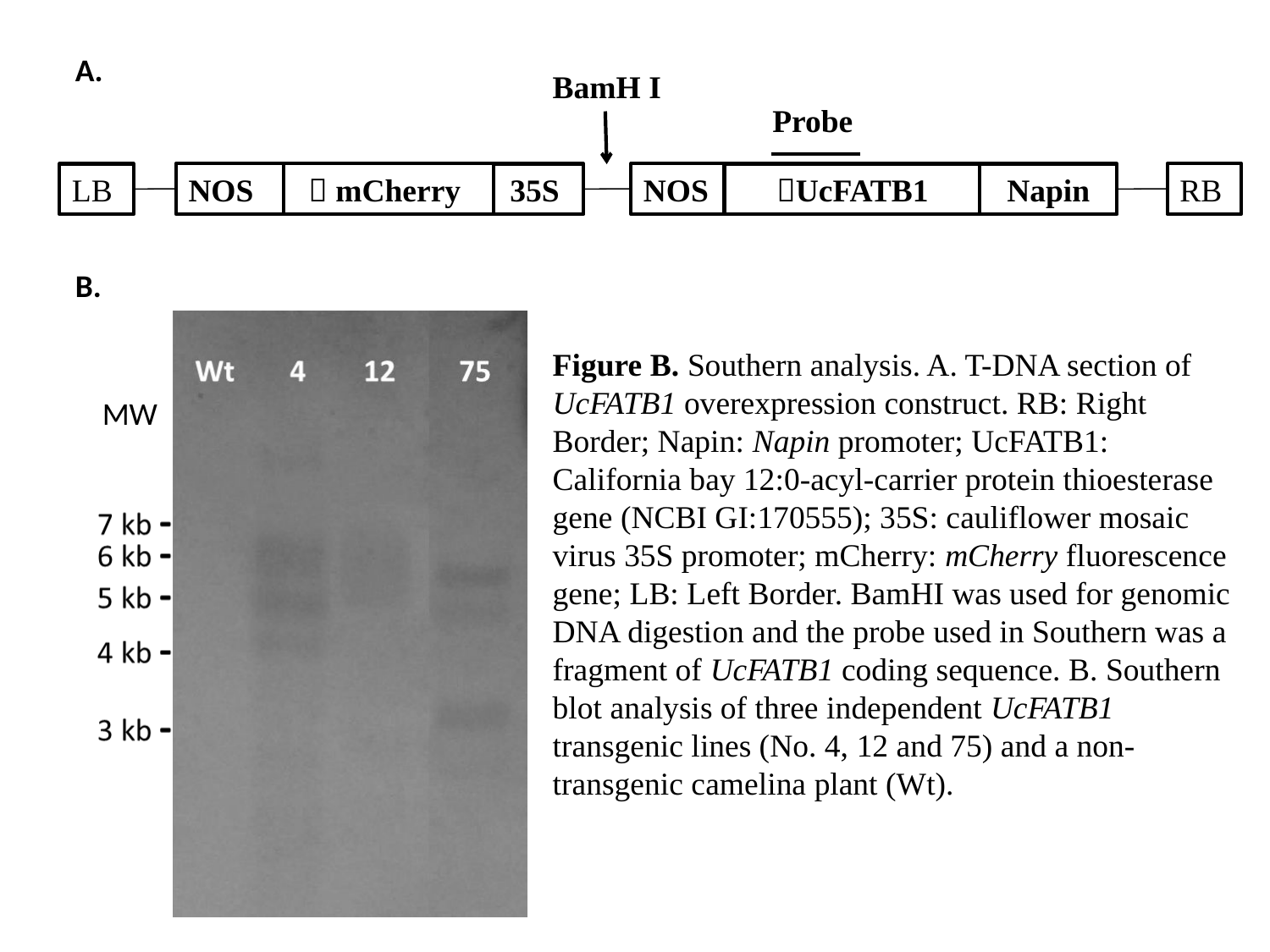

A.
BamH I
Probe
NOS
 mCherry
NOS
RB
LB
35S
UcFATB1
Napin
B.
Figure B. Southern analysis. A. T-DNA section of UcFATB1 overexpression construct. RB: Right Border; Napin: Napin promoter; UcFATB1: California bay 12:0-acyl-carrier protein thioesterase gene (NCBI GI:170555); 35S: cauliflower mosaic virus 35S promoter; mCherry: mCherry fluorescence gene; LB: Left Border. BamHI was used for genomic DNA digestion and the probe used in Southern was a fragment of UcFATB1 coding sequence. B. Southern blot analysis of three independent UcFATB1 transgenic lines (No. 4, 12 and 75) and a non-transgenic camelina plant (Wt).
MW

## Slide 5
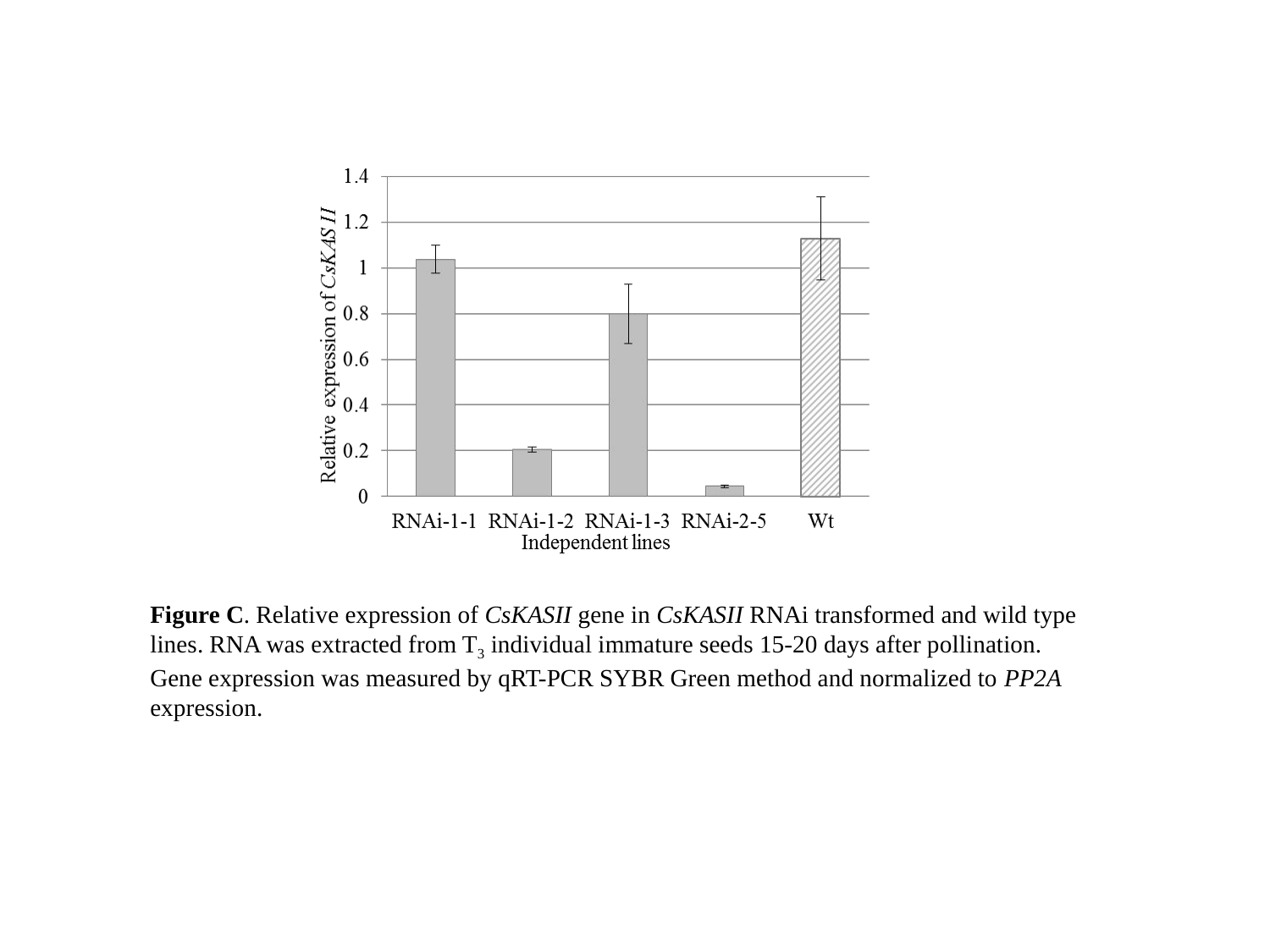

Figure C. Relative expression of CsKASII gene in CsKASII RNAi transformed and wild type lines. RNA was extracted from T3 individual immature seeds 15-20 days after pollination. Gene expression was measured by qRT-PCR SYBR Green method and normalized to PP2A expression.
